# A rare case of eosinophilic annular erythema, an uncommon new entity of eosinophilic dermatosis

**DOI:** 10.1002/ccr3.9009

**Published:** 2024-05-31

**Authors:** Saman Al‐Zahawi, Maryam Nasimi, Vahidesadat Azhari, Zahra Razavi

**Affiliations:** ^1^ Department of Dermatology Razi Hospital, Tehran University of Medical Sciences (TUMS) Tehran Iran; ^2^ Department of Dermatopathology Razi Hospital, Tehran University of Medical Sciences (TUMS) Tehran Iran; ^3^ Autoimmune Bullous Diseases Research Center Razi Hospital, Tehran University of Medical Sciences Tehran Iran

A 3‐year‐old boy was referred to Razi Dermatology Hospital due to multiple pruritic plaques on the trunk and proximal extremities for 2 months. His parent reported multiple, self‐healing, similar pruritic skin eruptions since 1 year ago. They denied any preceding pain or edema before skin eruptions. Physical examination revealed multiple annular and arcuate‐like erythematous plaques in the posterior neck, face, trunk, back, proximal, and distal lower extremities (Figure 1A–C). The central clearing was noticed in some of the lesions. Initial lab test for complete blood count, liver function, renal function, thyroid function, blood sugar, lactate dehydrogenase were within normal range. Complete blood count lacked leukocytosis and peripheral eosinophilia. A punch trunk biopsy was performed with the differential diagnosis including erythema annular centrifugum (EAC), subcutaneous lupus erythematous, urticaria vasculitis, granuloma annulare, erythema marginatum, and eosinophilic annular erythema (EAE). Histological evaluation showed the dermal infiltration of lymphocytes and numerous dermal eosinophils with a flame figure (Figure 2A–C). No signs of vasculitis, granulomatous changes, or blister formation were seen in the histopathological evaluation. The diagnosis of EAE was made depending on the clinical and pathological findings and the onset of the disease was attributed to more than 1 year ago when the child was only 2‐year‐old. An oral antihistamine, loratadine syrup, with topical triamcinolone acetate was started. Unfortunately, the patient did not come back for further evaluation and follow‐up.

**FIGURE 1:**
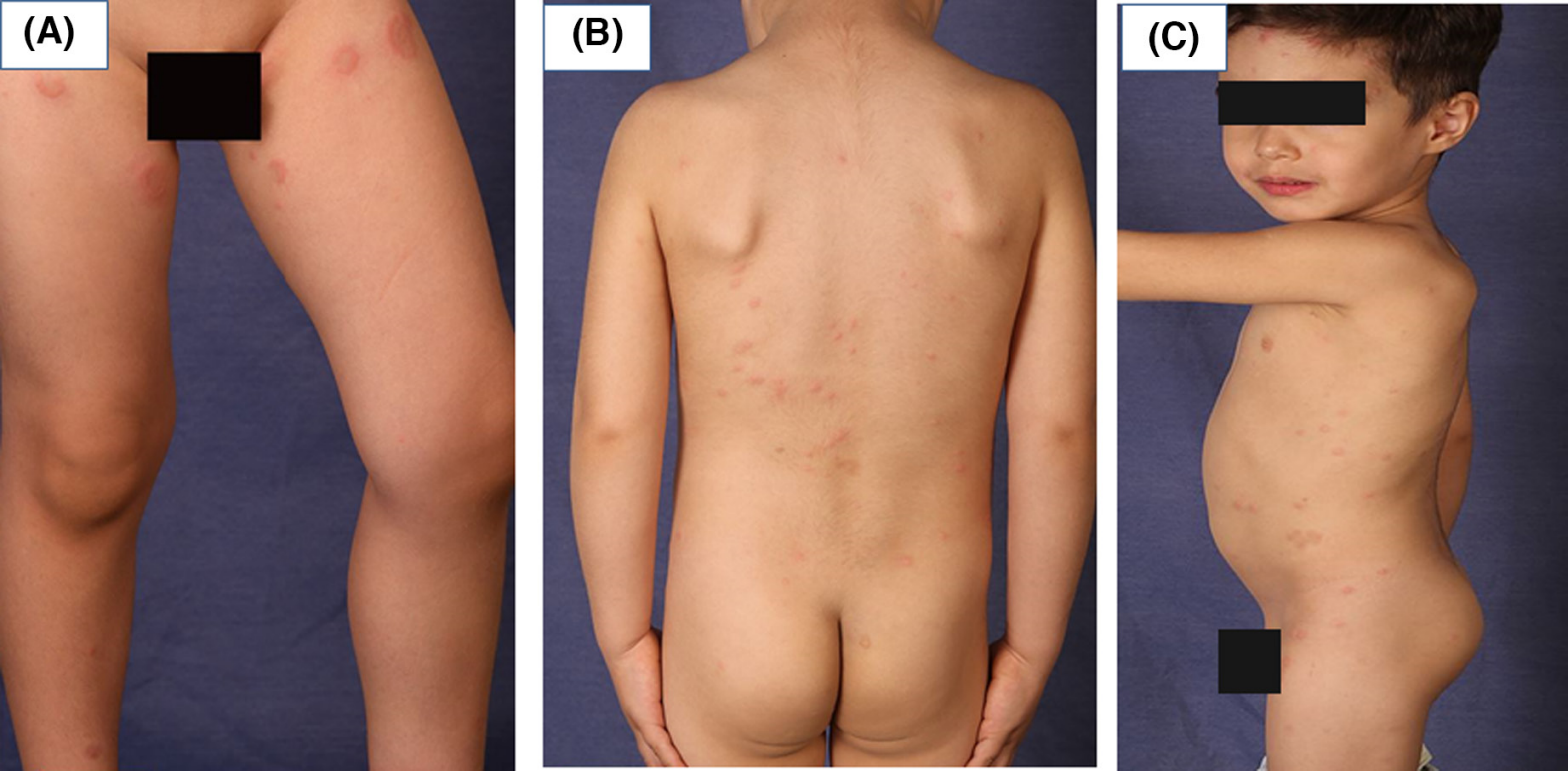
Multiple arciform and annular plaques with central clearing on the lower extremities (A) discrete erythematous plaques without scale in the back (B), and lastly, annular plaques involving the trunk and the face (C).

**FIGURE 2:**
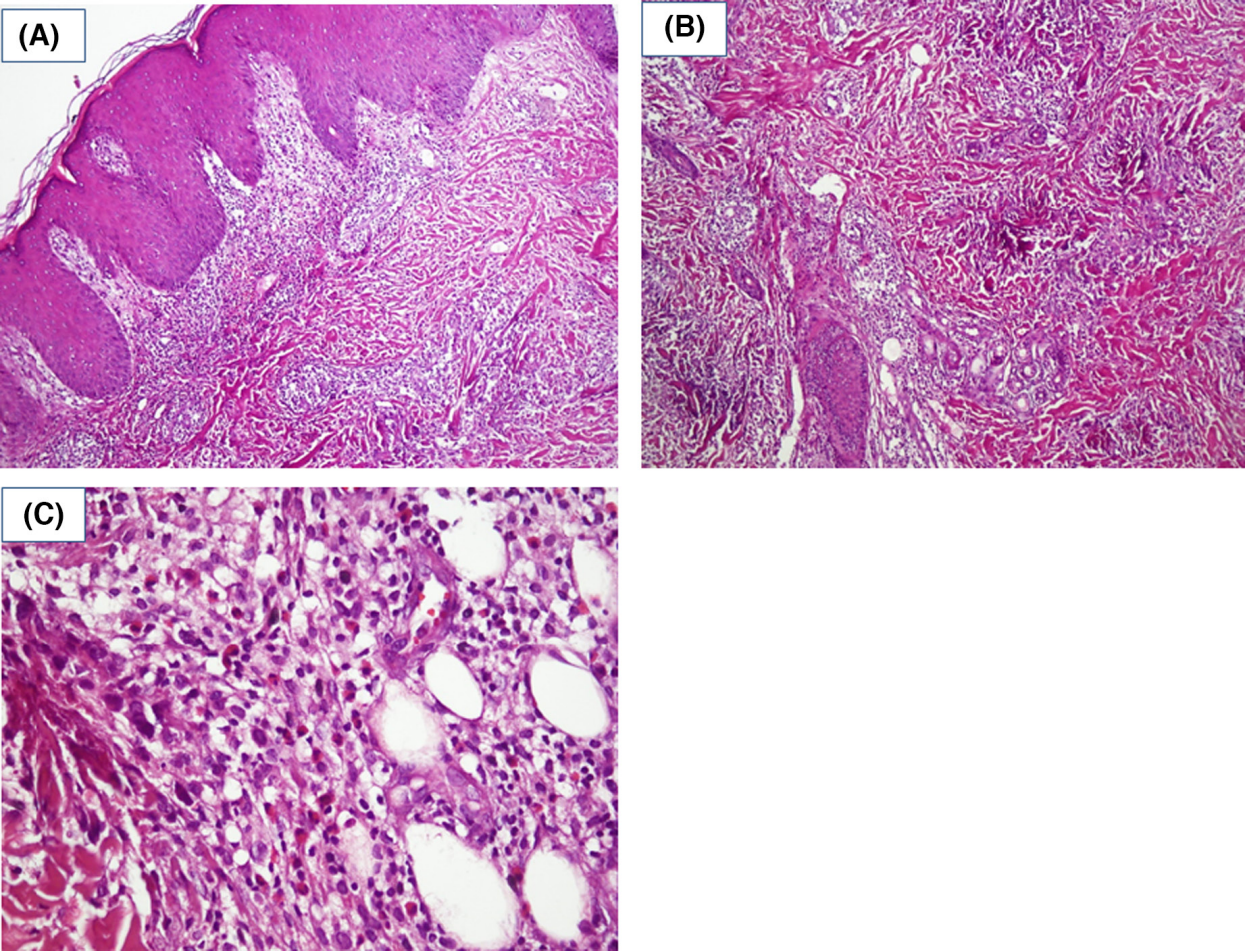
Histological pictures, Hemotoxin, and Eosin staining; Superficial interstitial and perivascular lymphocytic and eosinophilic infiltrate is seen 10× (A). Flame figure observed 10× (B). Deep dermal infiltrate consists of numerous lymphocytes and eosinophils 40× (C).

EAE is a rare eosinophilic rich, figurate erythematous dermatosis. EAE has no predilection for specific age group, but typically presents after the age of 1 year. The youngest reported case was 20‐month‐old and the eldest patient reported to be 82‐year‐old.^1^, ^2^ The precise etiology of EAE is not understood. Hypersensitivity to an unknown antigen was suggested. Also, a vague association with chronic borreliosis was reported. However, antibiotic therapy against borreliosis was not effective in clearing EAE. Other possible associations are autoimmune thyroid disorders, renal cell carcinoma, diabetes mellitus, chronic gastritis with Helicobacter pylori infection, chronic kidney disease, chronic hepatitis C virus infection, thymoma, clear cell renal carcinoma, and metastatic prostate adenocarcinoma.^3^, ^4^, ^5^

EAE presents as pruritic annular or arcuate plaques mimicking deep EAC or annular urticarial‐type plaques, without having scales. It predominantly involves the trunk and proximal extremities. Bullae are not a standard presenting feature. However, a case of bullous EAE with negative DIF and IIF was reported.^6^ Plaques may show central clearing. Individual lesions last for more than 24 h; they may be asymptomatic or may present with pruritus or rarely may be painful. Post‐inflammatory hyperpigmentation or fading erythema may persist after successful treatment, but with no residual scar remnants.^3^, ^7^ Histologically, a classical finding is the presence of a dense superficial, deep perivascular, and interstitial lymphocytic infiltration, In addition to eosinophilic infiltration. The flame figure is not a typical presentation, although it may be present in the longstanding lesions. Focal basal vacuolar damage and pigment incontinence may be present. Mucin deposition and vasculitis are absent. The principal differential diagnosis is the annular erythema of infancy and Wells syndrome. Other entities that should be differentiated are deep EAC, tumid lupus erythematous, Jessner lymphocytic infiltrate, urticarial phase of bullous pemphigoid, interstitial granulomatous disorders, and granuloma annulare.^3^, ^7^

Although EAE could be self‐healing, it usually follows a chronic relapsing and remitting course that is resistant to multidrug treatment. Systemic steroids and antimalarial (hydroxychloroquine) are the standard first‐line therapy that can clear the lesions. However, relapse after discontinuation is frequently reported.^3^, ^4^ Hydroxychloroquine combined with corticosteroid reported to be beneficial in refractory cases.^8^ Clinical remission with nicotinamide, cyclosporine, dapsone, indomethacin, doxycycline, mycophenolate mofetil, dupilumab and phototherapy also has been reported.^3^, ^4^, ^8^, ^9^ A 39‐year‐old male patient with diffuse EAE was successfully treated with doxycycline 100 mg/twice daily with substantial resolution of lesions within 3 months, complete clearance within 1 year, but with recurrence after cessation of the drug. Mycophenolate mofitel was reported to clear skin lesions of EAE in a patient with autoimmune hepatitis but was discontinued because of raised liver enzymes. Also, an eight sessions narrow‐band ultraviolet B phototherapy was reported to result in complete clearance of lesions of EAE in an 8‐year‐old male patient. Lastly, dupilumab was reported to successfully treat 14‐year‐old female patient with EAE.

## AUTHOR CONTRIBUTIONS

**Saman Al‐Zahawi:** Writing – original draft; writing – review and editing. **Maryam Nasimi:** Conceptualization; supervision. **Vahidesadat Azhari:** Supervision; visualization. **Zahra Razavi:** Supervision; writing – original draft; writing – review and editing.

## FUNDING INFORMATION

No funding was received for this article.

## CONFLICT OF INTEREST STATEMENT

None.

## CONSENT

Written informed consent was obtained from the patient's parents.

## Data Availability

The authors elect not to share data.

## References

[1] Paulitschke V , Tittes J , Tanew A , Radakovic S . Eosinophiles anuläres Erythem bei einem 20 Monate alten Mädchen. Hautarzt. 2021;72(4):332‐336. 32930857 10.1007/s00105-020-04687-z PMC8016794

[2] Ikutama R , Ogawa T , Takeno K , Ikeda S . A case of eosinophilic annular erythema (EAE) concomitant with autoimmune hypothyroidism. Indian Dermatol Online J. 2023;14(6):882‐883. 38099015 10.4103/idoj.idoj_558_22 PMC10718097

[3] Wallis L , Gilson RC , Gilson RT . Dapsone for recalcitrant eosinophilic annular erythema: a case report and literature review. Dermatol Ther (Heidelb). 2018;8:157‐163. 29222624 10.1007/s13555-017-0214-1 PMC5825319

[4] Thomas L , Fatah S , Nagarajan S , Natarajan S . Eosinophilic annular erythema: successful response to ultraviolet B therapy. Clin Exp Dermatol. 2015;40(8):883‐886. 25958878 10.1111/ced.12668

[5] Karataş Toğral A , Seçkin D . Eosinophilic annular erythema: a late but complete response to hydroxychloroquine. Australas J Dermatol. 2017;58(3):228‐230. 26768795 10.1111/ajd.12445

[6] Lachance M , Vallée S , Gagné É . Vesiculobullous eosinophilic annular erythema: a case report. SAGE Open Med Case Rep. 2023;11:2050313X231181024. 10.1177/2050313X231181024 PMC10288418 37359280

[7] Lee HS , Yang JY , Kim YC . Eosinophilic annular erythema localized to the palms and the soles. Ann Dermatol. 2016;28(6):769‐771. 27904280 10.5021/ad.2016.28.6.769 PMC5125962

[8] Chastagner M , Shourik J , Jachiet M , et al. Treatment of eosinophilic annular erythema: retrospective multicenter study and literature review. Ann Dermatol Venereol. Vol 149. Elsevier Masson; 2022:123‐127. 34716028 10.1016/j.annder.2021.07.007

[9] Young JN , Chung J , Fett N . Eosinophilic annular erythema responding to doxycycline. Cureus. 2023;15(10):e47478. 38022231 10.7759/cureus.47478 PMC10660796

